# Transcultural adaptation and validation of a French version of the University of California, Los Angeles geriatrics attitudes scale (UCLA-GAS-F)

**DOI:** 10.1186/s40945-021-00114-1

**Published:** 2021-09-07

**Authors:** Emmanuelle Opsommer, Odile Chevalley, Irene Pegito, Philippe Demeulenaere

**Affiliations:** School of Health Sciences (HESAV), University of Applied Sciences and Arts Western Switzerland (HES-SO), Avenue de Beaumont 21, 1011 Lausanne, Switzerland

**Keywords:** Attitudes, Elderly people, Cross-cultural adaptation, Reliability, Validity

## Abstract

**Background:**

In the context of demographic aging, active aging must be encouraged. In addition, the increase in life expectancy requires specific care for the elderly. Therefore, it is important to ensure appropriate training and education to caregivers. Educational institutions put value in positively influencing the attitudes and behaviours towards elderly people in order to ensure the quality of patient care in the future. Questionnaires are often used to assess attitudes. Among them, the University of California, Los Angeles Geriatrics Attitudes Scale (UCLA-GAS) was developed to assess attitudes towards older people and caring for older patients. This scale has been used to evaluate attitude of healthcare professionals and students including undergraduate physiotherapy students. To our knowledge, there is no scale that assesses the same concept in French. Therefore, this study aimed to translate and adapt the UCLA-GAS into French and to test its psychometric properties.

**Methods:**

We conducted this study in two phases. First, we translated and adapted the UCLA-GAS from English into French following the five recommended stages of cross-cultural adaptation. Second, we validated the French version of the scale with undergraduate physiotherapy students. One hundred nineteen students participated from the first to the third academic years. We estimated reliability and validity of the scale. We performed correlation analyses between the French version of the UCLA-GAS (UCLA-GAS-F) with the Aging Stereotypes and Exercise Scale (ASES) and the Attitudes to Aging Questionnaire (AAQ).

**Results:**

The scale was translated and adapted into French. Results of the validation phase showed that the UCLA-GAS-F had high test-retest reliability (ICC 0.83, 95%CI 0.74–0.89), but internal consistency below 0.7 (Cronbach’s alpha 0.49 to 0.57). The scale showed no ceiling or floor effect. As expected, the French version showed a weak correlation to the ASES (r = 0.28, *p* = .003) and to the AAQ (r = 0.32, *p* = .001).

**Conclusions:**

Despite low internal consistency, the French version of the UCLA-GAS showed appropriate psychometric properties. Further validation should include healthcare professionals and other healthcare students.

**Supplementary Information:**

The online version contains supplementary material available at 10.1186/s40945-021-00114-1.

## Introduction

Demographic aging can make a positive contribution to the community and society when policies and programs that encourage active aging are developed and implemented. Amongst four components necessary for a health-policy response, the WHO policy framework on active aging [1] recommended to provide training and education to caregivers [2]. Yet, important barriers such as stereotypes of aging can limit development of health-policy but can also restrain health-care professionals to work with older adults [3]. Nevertheless, the increase in life expectancy goes hand in hand with specific health care for the elderly and an increased demand for health professionals able to meet the needs of older people. Furthermore, in high-income countries, adults aged 60 years or older gave “health-care provider’s skills inadequate” (19.0%) and “previously treated badly” (23.8%) as frequent reasons for not accessing health-care services [2]. The aging of Switzerland’s population will accelerate rapidly between 2020 and 2030. Next to an increase in life expectancy, the population proportion aged 65 and over is estimated to increase from 18.9% in 2020 to 25.6% in 2050 [4].

Along with aging of populations, the prevalence of chronic diseases is rising quickly across countries [5–8]. Half of people (50.4%) aged 65 and over suffers from a chronic disease or a lasting health problem and 30.7% are limited in their activities for at least six months due to health problems in Switzerland [9]. Chronic symptoms in older people are significantly associated with disability in basic activities of daily living (Basic ADLs) and unfavourable quality of life (QoL) [7]. As a result, chronic diseases that are more prevalent in older adults increase the need for health care services [10].

With the population aging and the prevalence of chronic diseases, there is a need for specialized geriatric healthcare workers and training efforts in geriatrics and gerontology within educational institutions. To maintain or encourage better health and wellness, physiotherapists as experts in functional movement spur people of all ages to become more physically active by promoting, guiding, and managing physical activities and exercises. Physical activity is highly recommended in presence of chronic symptoms (i.e., joint pain and back pain) [7, 11, 12]. Indeed, physical inactivity and sedentary behaviours are modifiable risk factors on which the physiotherapist can act [13]. To ensure that physiotherapists as part of a multidisciplinary team can play their role when working with older people, the initial and continued education of physiotherapists need to promote high standards in physiotherapy with older people.

Hence, for educational institutions, positively influencing attitudes and behaviours towards older people is particularly important to ensure the quality of patient care in the future. Indeed, older adults currently represent at least 40% of the clinical caseload for current health-care students [14, 15]. Educational interventions increase knowledge and skills and can improve health care student attitudes and behaviours towards older adults. Various approaches are used, which differ in their effectiveness to foster positive attitudes and behaviours towards older patients [14, 16]. For instance, interventions that focus on improving knowledge of students about aging or older patients effectively increased knowledge but were unsuccessful at improving positive attitudes towards older patients [17].

To give rise to positive change in student attitudes towards older adults, a knowledge-directed geriatrics intervention should strive to foster empathy as well by encouraging students to share experiences with older adults outside the clinical setting [17]. Indeed, interventions incorporating interactions with older patients who were independently living and high functioning improved student attitudes towards older adults post intervention in comparison to control groups [16].

Studies that attempted to change attitudes towards older adults often used questionnaires to measure attitudes. A wide array of quantitative measurement tools assess views on aging or attitude towards older people [18, 19] and can differentiate students who have or have not had educational intervention designed to improve health care student attitudes towards older people [16]. Amongst them, the University of California, Los Angeles Geriatrics Attitudes Scale (UCLA-GAS) [20] is one of the most widely used and cited assessment instruments [18, 21]. This short instrument (14 items) measures general attitudes towards older people and caring for older patients. The scale was developed with primary care residents, geriatrics fellows, and geriatrics faculty [20] and has also been used to evaluate the attitude of other health professionals [20, 22] as well as students [21, 23–26] including undergraduate physiotherapy students [21, 23].

To our knowledge, there is no self-report assessment tool of attitude towards older people translated and validated for French-speaking countries. The availability of such a tool in French investigating healthcare professionals’ attitudes towards older people and caring for older patients may be of particular interest and usefulness in French-speaking educational and clinical institutions. Therefore, this study aimed to translate and adapt the UCLA-GAS into French and test its psychometric properties.

## Methods

We conducted this validation study in two phases. In phase I, we translated and adapted the UCLA-GAS from English into French. In phase II, we performed the initial validation of the French version of the UCLA-GAS (Table 1).

**Table 1:** French version of the University of California, Los Angeles Geriatrics Attitudes Scale (UCLA-GAS-F)

Item	UCLA-GAS	French version UCLA-GAS
	DIRECTIONS: Please use the scale to indicate the degree to which you agree or disagree with each statement. There are no right or wrong answers. The best response is the one that truly reflects your personal opinion. Findings of this study will be reported only on a group basis with no individual names identified. “Old people” and “elderly patients” mentioned in the questions refer to persons aged 65 or older.	INDICATIONS: Veuillez utiliser l’échelle pour indiquer dans quelle mesure vous êtes d’accord ou pas avec chaque énoncé. Il n’y a pas de bonnes ou de mauvaises réponses. La meilleure réponse est celle qui reflète vraiment votre opinion personnelle. Les résultats de cette étude seront rapportés uniquement par groupe, sans noms individuels identifiés. Les « personnes âgées » et les « patients âgés » mentionnés dans les questions se réfèrent aux personnes âgées de 65 ans et plus.
1	Most old people are pleasant to be with.	La plupart des personnes âgées sont d’agréable compagnie.
2	The federal government should reallocate money from Medicare to research on AIDS or pediatric diseases.	Le gouvernement (fédéral) devrait réallouer des fonds de l’assurance-maladie des personnes âgées à la recherche sur le VIH ou les maladies pédiatriques.
3	If I have the choice, I would rather see younger patients than elderly ones.	Si j’ai le choix, je préfère voir des patients jeunes plutôt que des patients âgés.
4	It is society’s responsibility to provide care for its elderly persons.	Il est de la responsabilité de la société de fournir des soins aux personnes âgées.
5	Medical care for old people uses up too much human and material resources.	Les soins médicaux aux personnes âgées mobilisent trop de ressources humaines et matérielles.
6	As people grow older, they become less organized and more confused.	Lorsque les personnes vieillissent, elles deviennent moins organisées et plus confuses.
7	Elderly patients tend to be more appreciative of the medical care I provide than are younger patients.	Les patients âgés ont tendance à être plus reconnaissants des soins médicaux que je leur prodigue que les jeunes patients.
8	Taking a medical history from elderly patients is frequently an ordeal.	Recueillir les antécédents médicaux auprès des personnes âgées est souvent pénible.
9	I tend to pay more attention and have more sympathy towards my elderly patients than my younger patients.	J’ai tendance à porter plus d’attention et éprouver plus de sympathie envers mes patients âgés qu’envers mes patients plus jeunes.
10	Old people in general do not contribute much to society.	En général, les personnes âgées ne contribuent pas beaucoup à la société.
11	Treatment of chronically ill old patients is hopeless.	Le traitement des patients âgés atteints de maladies chroniques est vain.
12	Old persons don’t contribute their fair share towards paying for their health care.	Les personnes âgées ne contribuent pas de manière équitable aux coûts de leurs soins de santé.
13	In general, old people act too slow for modern society.	En général, les personnes âgées agissent trop lentement pour la société actuelle.
14	It is interesting listening to old people’s accounts of their past experiences.	Il est intéressant d’écouter le récit que les personnes âgées font de leurs expériences passées.

*UCLA-GAS*: University of California, Los Angeles Geriatrics Attitude Scale

### Phase 1: translation and cross-cultural adaptation of UCLA-GAS into French

For the cross-cultural adaptation of the UCLA-GAS, we asked permission from the developers of the scale [20] and followed the process based on Beaton and collaborators [27].

Two translators worked separately to provide two independent forward translations (Tr1 and Tr2) from the original language (English) to the target language (French) and then worked together to produce a consensus translation (Tr-12). The target language was the first language for each translator. Both translators each produced a written report of the translation that they completed with the rationale for their choices. The consensus translation (Tr-12) was submitted to a linguist, discussed to identify poor wording choices, and resolved in a discussion between the linguist and translators.

We sent the Tr-12 version of the questionnaire to two persons with the source language (English) as their mother tongue. They were uninformed of the concepts explored and blinded of the original questionnaire; they produced two independent back-translations (BT1 and BT2) with a written report of their translation.

Finally, a committee including health professionals, methodologists and the translators met to review all the versions of the questionnaire and to develop the pre-final version of the questionnaire for field-testing. We appraised equivalence between the source and target versions in four areas (semantic, idiomatic, experiential and conceptual equivalences) [27].

### Phase 2: psychometric validation of the French version of the UCLA-GAS

For initial validation of the questionnaire, we administered the questionnaire to physiotherapy undergraduate students who wanted to participate in the study and who were present when the questionnaire was administered. We estimated reliability (internal consistency and test-retest reliability) and construct validity (discriminant and structural). To provide external measures, we compared the UCLA-GAS-F to the Aging Stereotypes and Exercise Scale (ASES) initially developed in French [28] and seven selected items, which can be addressed by a large public, of the French version of the Attitudes to Aging Questionnaire (AAQ) [29]. The local Ethics Committee approved the study (Req-2018-00467) according to the Swiss law [30].

#### Study participants

The French version of the UCLA-GAS (UCLA-GAS-F) was administered to 157 undergraduate students at the School of Health Sciences in Lausanne (HESAV). From the 157 registered students, 119 responded at both assessment times. The groups comprised of undergraduate physiotherapy students of all grades, from the first to the third academic year. A minimum sample size of 70 was estimated based on the recommendation by the Consensus-based Standards for the Selection of Health Measurement Instruments (COSMIN) [31]. It recommends at least 5 to 10 participants per item.

#### Data collection tools

Participants completed twice (T1 and T2) the UCLA-GAS-F with one-week interval to assess test-retest reliability. The interval was considered long enough to avoid recall bias and short enough to avoid changes due to training or time [32]. In addition, they completed the ASES (developed in French) and seven selected items of the French version of the AAQ to assess construct validity at T1.

The UCLA-GAS is a one-dimension questionnaire to assess health care providers’ attitudes towards older persons and caring for older patients [20]. The scale contains 14 items assessed on a 5-point Likert scale ranging from 1 (strongly disagree) to 5 (strongly agree) with a score of 3 indicating a neutral response (Table 1). Five items (Table 1: items 1, 4, 7, 9 and 14) are positively worded (e.g., “I tend to pay more attention and have more sympathy towards my elderly patients than my younger patients”) and nine are negatively worded (e.g., “Old persons don’t contribute their fair share towards paying for their health care”). Scores of the negatively worded items are reversed to calculate the total score that can range from 14 to 70. A higher score indicates a positive attitude of participants towards older persons and a score of 42 indicates a neutral attitude. The original version of the scale showed no floor or ceiling effect and adequate internal consistency (Cronbach’s α = 0.76) [20]. Its validity was assessed by construct validity and known-groups validity. For construct validity, the scale demonstrated adequate convergent correlations (Pearson correlation, *r* = 0.58, *p* < 0.001) with two subscales of the Maxwell-Sullivan Scale (“Too much time to care” and “No benefit of treatment”). In addition, it demonstrated low correlation with geriatrics knowledge (Pearson correlations, *r* = 0.07 and *r* = 0.26, in the initial and cross validation studies [20]). For known-groups validity, the scale was able to differentiate between first- and second-year residents with geriatrics faculty and fellows, and between residents with different career interests. The scale also showed sensitivity to change. Indeed, residents and fellows followed over a 2-year period showed a significant increase in attitude scores [20]. In addition, a factor analysis revealed four different components of the scale: Perceived Social Value of older people, Medical Care provided to geriatric patients, Compassion towards older people, and Distribution of Societal Resources for older people [22]. For the overall scale, the internal consistency was 0.78 but much less for the subscales (0.60–0.62) [22].

Besides the UCLA-GAS-F, the participants completed the ASES and AAQ. The ASES aims to measure dimensions of exercise-related aging stereotypes in general population [28]. This 12-item scale is subdivided in three subscales of four items each: Risk of exercise, Benefits of exercise and Psychological barriers. Each item is assessed on a 7-point Likert scale ranging from 1 (do not agree at all) to 7 (totally agree). To obtain a total score for the scale, the score of each item from the Risk of exercise subscale (items 3, 6, 9 and 12) must be reversed, and then added to the scores of the other subscales. The total score ranges from 12 to 84. A high score shows positive stereotypes regarding exercise and aging. The three subscales demonstrated satisfactory reliability (Cronbach’s alpha of 0.84, 0.87 and 0.84 for Psychological barriers, Benefits of exercise, and Risks of exercise subscales respectively). The test-retest reliability was adequate (Benefits of exercise subscale: *r* = 0.57, Risks of exercise subscale: *r* = 0.59, and Psychological barriers subscale *r* = 0.53). Good fit to the data of invariance of the factorial structure across age suggests that the factorial structure of the scale was similar across age [28].

The AAQ was developed by international experts from the World Health Organization Quality of Life (WHOQOL) Group in 2007 [33]. It aims to assess attitudes towards the aging process as a personal experience from the perspective of older adults. It is multidimensional and can be used in cross-cultural settings. The questionnaire focuses on three different aspects of aging: Psychosocial Loss, Physical Change, and Psychological Growth. The questionnaire consists of 24 items, with eight items for each subscale. Each item is assessed on a five-point Likert scale, ranging from 1 (strongly disagree or not at all true) to 5 (strongly agree or extremely true). Scores range from 8 to 40 on each subscale. Higher scores on the Physical Change and the Psychological Growth subscales show a more positive appraisal of one’s own aging, whereas higher score on the Psychosocial Loss subscale shows a more negative appraisal. A total score can also be obtained on all 24 items (after the scores of the Psychosocial Loss subscale are reversed). Higher total scores indicate larger positive attitudes towards one’s own aging process. The scale was assessed for validity and reliability in French and demonstrated acceptable psychometric qualities [29]. We selected the first seven items of the scale as they are more general and can be addressed by a large public. The other items are addressed for people over 60 years old. The items selected were: 1) “As people grow older they are better able to cope with life”, 2) “It’s a privilege to grow older”, 3) “Old age is a time of loneliness”, 4) “Wisdom comes with age”, 5) “There are many pleasant things about growing older”, 6) “Old age is a depressing time of life”, and 7) “It is important to take exercise at any age”.

#### Data analyses

We extracted data from the paper questionnaires and analysed anonymised data with SPSS version 25 [34]. The scores were determined following the guidelines of the instruments. When missing values occurred, we assessed the proportion and the mechanism of missing data [35, 36] for each scale. Due to the limited amount of missing data (below 5%), we performed complete-case analysis [35].

Demographic data (age, gender, year of study, and origin) of the participants were retrieved at T1. Data were tested for normal distribution with Kolmogorov-Smirnov test and for homoscedasticity with Levene test. For descriptive analysis, mean and standard deviation (SD) were calculated for normally distributed data for all participants, and for each study year (1st year, 2nd year and 3rd year Bachelor degrees). The score on the UCLA-GAS-F were compared between gender with t-test and between year of study with one-way ANOVA.

Test-retest reliability that refers to ”the extent to which scores for patients who have not changed are the same for repeated measurement over time” [37] was assessed with the intraclass correlation coefficient with two-way random effect model for absolute agreement (ICC model_2,1_ agreement) [38]. An ICC ≥0.7 reflects good reliability [31]. We assessed the agreement between assessment times (T1 and T2) with the Bland-Altman method. We calculate the 95% limits of agreement using the mean and the standard deviation of the differences between the two measurements and we plotted a Bland Altman plot [31]. To report test-retest reliability on each unidimensional score, a Cohen’s kappa was calculated for each item of the scale, with the interpretation that a value ≥0.8 is almost perfect, ≥ 0.6 is substantial, ≥ 0.4 is moderate, ≥ 0.2 fair, and below 0.2 is slight [39].

The internal consistency that reflects the extent to which the items are inter-correlated was estimated using the Cronbach’s alpha coefficient [40]. Good internal consistency is considered with a Cronbach’s alpha coefficient between 0.70 and 0.95 [41].

Construct validity was tested with discriminant validity which suggests that the score of one scale should not be highly correlated with the score from another scale assessing a construct that theoretically should not be highly related. Hypotheses on the correlations between the UCLA-GAS-F and the ASES and AAQ were defined a priori. A poor correlation was expected between UCLA-GAS-F and ASES as the first scale measures attitude towards elderly people in the health system whereas the second measures attitude towards physical activity in elderly people. Poor to fair correlation was expected between UCLA-GAS-F and AAQ. Pearson correlation was used to assess the correlation between the results of the UCLA-GAS-F, the ASES and the AAQ at T1. Poor, fair, moderate and very strong correlations were considered for Pearson’s r of < 0.3, 0.3–0.5, 0.6–0.8, and > 0.8 respectively [42].

In addition, we conducted a confirmatory factor analysis (CFA) to test the structure proposed in the original version [22]. To test the model, we used a weighted-least squares estimator with robust estimation of means and variances (WLSMV) [43]. To quantify the degree of fit of the model, we reported a chi-square test (χ^2^) and the following indices [44]: the comparative fit index (CFI) which is an incremental fit index that measures the proportionate improvement fit and two absolute fit indices that assess how well a-priori model reproduces the sample data. The latter are the root mean square error of approximation (RMSEA) and the standardized root mean square residual (SRMR). CFI values > 0.95 represent very good fit. RMSEA values ≤0.05 represent very good fit and between 0.05–0.08 good fit. SRMR values < 0.08 represent good fit [44]. Further, factor loadings (λ), which are the patterns of relationship between the common factors and the indicators [43], are reported. CFA was performed with R Program [45], version 4.1.0, with the lavaan package [46].

*P* values of < 0.05 were considered significant.

## Results

### Phase 1: translation and cross-cultural adaptation of UCLA-GAS into French

The UCLA-GAS scale was translated and adapted into French (Table 1 and Supplementary material). Equivalence of the different translations, back-translations and original scales was confirmed during the expert committee. Translation of item 2 required attention for adaptation as “Medicare”, a specific American federal insurance for people over 65 years old, does not have equivalent in Switzerland. Discussion about this item was necessary. In the French version, item 4 was translated without maintaining the possessive pronoun before society, whereas the possessive pronoun was used in the French version for the item 9 as it was in the English version.

### Phase 2: psychometric validation of the French version of the UCLA-GAS

#### Characteristics of participants

One hundred nineteen of the 157 students participated in the study, namely a response rate of 75.8% (47/58 in the first year, 43/50 in the second year and 29/49 in the third year). Two-thirds (67.2%) of the participants were women. Mean age of the participants was 24.15 (SD 3.06) years old. Participants were mainly of Swiss nationality (87.4%) or other European nationalities (12.6%). Namely, nine participants were French, two Portuguese, one Spanish, one Italian, one British and one Irish. Their mother-tongue was French for all of them but one.

#### Attitudes of undergraduate physiotherapy students towards elderly people

In Table 2, mean total scores for the UCLA-GAS-F, ASES and AAQ at T1 and UCLA-GAS-F at T2 are reported.

**Table 2:** Scales and subscales scores for each academic year

Scales (min – max total scores)	All participants (*n* = 119) *Mean (SD)*	1st year participants (*n* = 47) *Mean (SD)*	2nd year participants (*n* = 43) *Mean (SD)*	3rd year participants (*n* = 29) *Mean (SD)*
**UCLA-GAS-F at T1** (14–70)	48.50 (4.23)	47.37 (4.12)	49.28 (4.37)	49.18 (3.90)
Perceived Social value (2–10)	7.16 (1.48)	6.85 (1.52)	7.44 (1.26)	7.24 (1.68)
Medical care (4–20)	13.48 (2.17)	12.80 (2.14)	13.70 (1.99)	14.24 (2.21)
Compassion (4–20)	13.74 (1.80)	13.85 (1.86)	13.60 (1.63)	13.76 (1.98)
Distribution of Societal Resources (4–20)	14.21 (1.72)	13.91 (1.59)	14.53 (1.96)	14.21 (1.50)
**UCLA-GAS-F at T2** (14–70)	48.52 (4.80)	48.67 (3.91)	48.15 (5.66)	48.86 (4.77)
**ASES** (12–84)	65.66 (6.54)	62.24 (6.19)	67.73 (6.50)	68.03 (4.76)
Benefits stereotype (4–28)	24.08 (2.87)	22.91 (2.90)	24.77 (2.74)	24.93 (2.45)
Risks stereotype (4–28)	24.76 (2.99)	23.47 (3.47)	25.07 (2.52)	26.31 (1.71)
Psychological barriers stereotype (4–28)	16.86 (3.10)	15.91 (2.85)	17.93 (3.47)	16.79 (2.40)
**AAQ** modified (7–35)	25.07 (2.81)	24.70 (3.02)	25.73 (2.59)	24.72 (2.71)

*Min* – *max total scores* = minimum and maximal total scores for each scale or subscale; *n* = number of participants; *SD* = standard deviation; *UCLA-GAS-F* = University of California, Los Angeles Geriatric Attitude Scale in French; *T1* = first evaluation; *T2* = second evaluation, *ASES* = Aging Stereotypes and Exercise Scale; *AAQ* = Attitudes to Aging Questionnaire

The mean score of the UCLA-GAS-F was in the positive range at T1 (48.50, SD 4.23, range 38–59) and at T2 (48.52, SD 4.80, range 34–63). No participant answered in a completely negative (1 or 2) or positive (4 or 5) way to all items of the UCLA-GAS-F; there were no floor or ceiling effects in our data. There was no significant difference between women and men (*p* = 0.235, mean total score 48.83, SD 4.07 and 47.85, SD 4.50 respectively). Mean UCLA-GAS-F score was smaller for first year BSc physiotherapy students (47.37, SD 4.12) than for second- and third-year students (49.28, SD 4.37 and 49.18, SD 3.90 respectively). However, the difference was not significant (*p* = 0.064).

The mean score of ASES was 65.66 (SD 6.54, range 51–84) and of AAQ 25.07 (SD 2.81, range 17–31). Mean ASES score was significantly smaller for first year BSc physiotherapy (62.24, SD 6.19) than for second and third years (67.73, SD 6.50 and 68.03, SD 4.76, respectively) (*p* < 0.001). No significant difference in mean total score was shown for the seven items of the AAQ between the three academic years (*p* = 0.187).

**Fig. 1:**
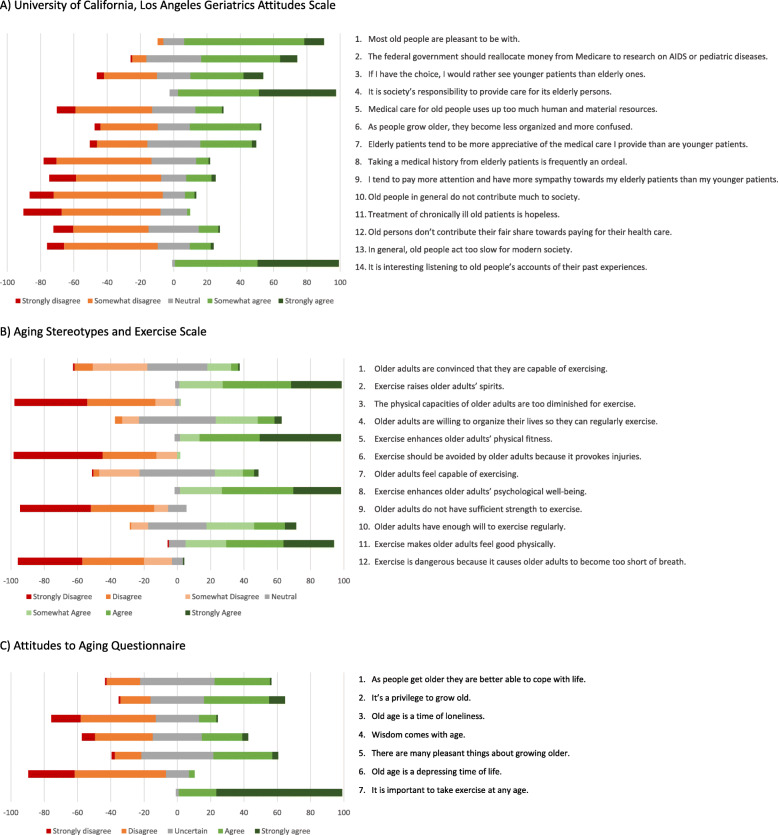
Frequency of responses for the French version of the University of California, Los Angeles Geriatrics Attitudes Scale (UCLA-GAS-F) from all participants for each item of the scale. In the figure, the red (strongly disagree) and orange (somewhat disagree) intervals from −100 to 0 represent the percentage of disagreement with the statements on the right. The light green (somewhat agree) and dark green (strongly agree) intervals from 0 to 100 represent the agreement with the statements. The neutral position is represented with grey intervals. Panel A stands for UCLA-GAS-F, panel B for Aging Stereotypes and Exercise Scale (ASES), and panel C for seven items of the Attitudes to Aging Questionnaire (AAQ)

The Fig. 1 shows percentage of agreement and disagreement for each item of the three scales. For the UCLA-GAS-F (Fig. 1A), the majority of students agreed with the following items: “Most old people are pleasant to be with” (84.0%), “It is society’s responsibility to provide care for its elderly persons” (95.0%), and “It is interesting listening to old people’s accounts of their past experiences” (98.3%). Furthermore, most of the students disagreed with the negatively worded statements: “Old people in general do not contribute much to society” (79.8%), and “Treatment of chronically ill old patients is hopeless” (82.2%).

#### Reliability and validity of the French version of the UCLA-GAS

**Fig. 2:**
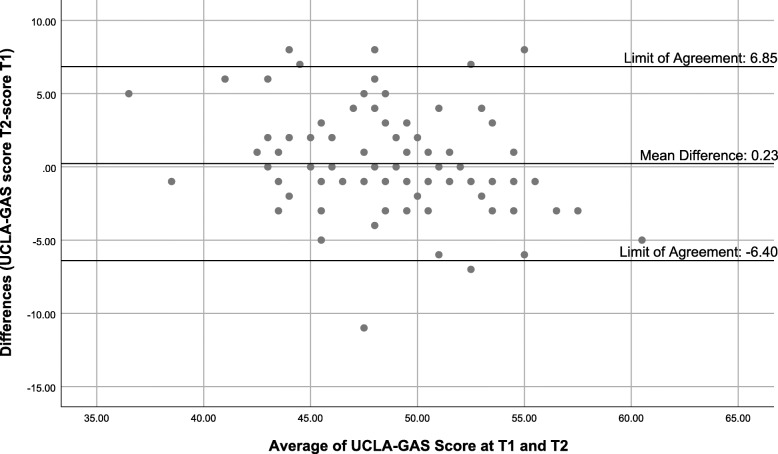
Bland-Altman plot for French version of the University of California, Los Angeles Geriatrics Attitudes Scale (UCLA-GAS-F) total score, with limit of agreements intervals of the mean difference between the two assessments

The UCLA-GAS-F showed good test-retest reliability between T1 and T2, with an ICC of 0.83 (95%CI 0.74–0.89). The limits of agreement from the Bland-Altman plot (Fig. 2) were − 6.4 to 6.85 for the UCLA-GAS-F. The mean difference between mean total scores at T1 and T2 was 0.23. The value for Cohen’s kappa varied between 0.24 and 0.52 demonstrating fair to moderate test-retest reliability of each individual item.

The internal consistency of the French version of the scale was lower than cut-off value. The Cronbach’s alpha coefficient was 0.49 for T1 and 0.57 for T2. The alpha coefficient would increase if any of the following items were removed: item 2 (to 0.52 at T1, and 0.61 at T2), item 4 (to 0.50 at T1, and 0.57 at T2), item 7 (to 0.54 at T1, and 0.59 at T2), and item 9 (to 0.56 at T1, and 0.61 at T2).

The validity of the scale was tested with the discriminant validity. Pearson correlation showed poor and fair correlations between ASES (r = 0.28, *p* = 0.003) and AAQ (r = 0.32, *p* = 0.001) with the UCLA-GAS-F.

For the structural validity, we used a CFA to determine if the model of Lee et al. [22] was appropriate to our data for the UCLA-GAS-F. We could not confirm the model with our sample data. All the overall goodness-of-fit indices suggest that the 4-factor model did not fit the data well: χ^2^ (71) = 135.653 (*p* < .001), SRMR = 0.098, RMSEA = 0.089 (90% CI = 0.066–0.111, *p* = 0.004), and CFI = 0.834. The factor loadings (λ) were low to moderate showing no strong relation between the items and the respective factors. For the factor “Perceived Social Value of older people”, the standardized factor loadings ranged from 0.658 (item 12) to 0.781 (item 13). For the factor “Medical Care provided to geriatric patients” composed of four items, the standardized factor loadings ranged from 0.235 to 0.694 with item 8 (λ = 0.317), item 6 (λ = 0.381), item 3 (λ = 0.235), and item 11 (λ = 0.694). The standardized factor loadings for “Compassion towards older people” were for item 7 (λ = 0.555), item 9 (λ = 0.639), item 1 (λ = − 0.195), and item 14 (λ = − 0.357). For “Distribution of Societal Resources for older people”, a factor with four items, the standardized factor loadings were for item 2 (λ = 0.022), item 4 (λ = 0.168), item 5 (λ = 0.467) and item 10 (λ = 0.609). From the 14 items, three items showed non-significant *p*-value for their factor loadings, item 1 (*p* = 0.094), item 2 (*p* = 0.806), and item 4 (*p* = 0.081).

## Discussion

The present study was designed to provide a French version of a self-report scale, UCLA-GAS-F, to measure attitudes towards older people and caring for older patients.

The results of this study on undergraduate physiotherapists’ attitudes towards older people indicate that the French translation of the UCLA-GAS has good test-retest agreement and reliability for use in educational institutions. The results apply to the French version and cannot be generalised to other languages. To our knowledge, no previous study explored the test-retest reliability of the original instrument version. In a recent review of self-reported measures of views on aging by Klusmann et al. [18], test-retest reliability was only available for 18% of the tools (*n* = 16/89) in comparison to the internal consistency which was reported for the vast majority (79%) of instruments (*n* = 70/89) including UCLA-GAS. Nevertheless, but using another approach than ICC, Sahin et al. [21] assessed the test-retest reliability of the Turkish version of the UCLA-GAS with a Pearson correlation analysis (r = 0.51; two weeks interval measurements on 120 health care providers).

Concerning internal consistency findings, we estimated a weaker value (Cronbach’s alpha < 0.7) to that achieved in the original publications [20, 22] and other studies with medical students [24, 47]. Nevertheless, in the literature, internal consistency findings are mixed, and several other studies scored below 0.7 [21, 48–52]. For this translated version, the internal consistency, which is an important measurement property for questionnaires, was not sufficient. As the homogeneity of the sample negatively influences Cronbach’s alpha, which was the case in the present study with only undergraduate physiotherapy students, future studies should be based on samples that are more heterogeneous with students from other disciplines and various health professionals. Indeed, a higher value of Cronbach’s alpha can be found in heterogeneous populations than in homogeneous populations [40]. Furthermore, the Cronbach’s alpha can be influenced by the number of items in the scale [40]. The analysis showed that the value of Cronbach’s alpha would be increased when item 2, 4, 7 or 9 is removed. However, to provide comparability with the original scale and because it was already a short questionnaire, we did not remove any item in the French version.

We attempted to replicate the original factor structure [22] but like other previous studies, we could not regenerate the 4-factor solution found in that study. Lee et al. [22] proposed a 4-factor structure of the UCLA-GAS based on a principal component analysis (PCA). This 4-factor structure did not fit the data of the current study as shown by the inconclusive goodness-of-fit indices. In addition, three items showed not significant factor loadings indicating an inappropriate fit to the model. However, we should be careful with the interpretation of these results because of the sample size. Nevertheless, contradictory results on the factor structure are reported in further studies. One study [53] could not regenerate the 4-factor structure with a factor analysis regardless of the factor rotation method used. Another study [47] identified a 4-factor structure but this CFA had only one factor (Compassion) that matched the initial structure. In addition, there was one additional item in this factor (in total five items) compared to the four items of the Compassion dimension by Lee et al. [22]. Only one study with the scale in Turkish confirmed the 4-factor structure with a PCA [21]. These contradictory results suggest that further research is needed to define the structure of the UCLA-GAS.

In order to estimate the validity of UCLA-GAS-F, there is no scale translated into French (valid and reliable) that measures exactly the same construct. Therefore, we compared the UCLA-GAS-F scale to scales measuring other constructs (i.e. exercise-related aging stereotypes (ASES) and attitudes towards the aging process as a personal experience (AAQ)). We confirmed our hypotheses of poor correlation of these scales with the UCLA-GAS-F. Other previous studies have compared the scale to scales based on the same construct (convergent validity). For instance, Reuben et al. [20] compared the scale they developed to two subscales of the Maxwell-Sullivan Attitude Survey and determined an adequate correlation (r = 0.58). The UCLA-GAS, however, showed poor correlation (r = 0.083) with the Carolina Opinions On care of Older Adults (COCOA), a survey to measure medical and other health professional students’ attitudes towards older adults and towards a career choice in geriatrics [47]. Furthermore, the UCLA-GAS was also shown to measure different construct than geriatrics knowledge as shown by discriminant validity with the geriatrics knowledge scores (r = 0.07 and r = 0.26) [20] and with the Revised Fact on Aging Quiz (r = − 0.04) [49].

In this study, the results of the UCLA-GAS-F indicated a positive attitude towards older persons and caring for older patients (mean score 48.50, SD 4.23) of the undergraduate physiotherapy students. Similarly, physiotherapy and rehabilitation students in Turkey showed positive attitude towards elderly people with the UCLA-GAS (mean score 48.18, SD 5.67) [23]. Further studies using the UCLA-GAS showed positive attitude towards elderly people in different health professional groups. Indeed, positive attitudes were observed with the UCLA-GAS for medical students [24, 26, 49], for students from different health care programs, including medicine, nursing, pharmacy and social work [48, 50], for medical students, residents and geriatrics fellows [51], for primary care residents and fellows [20, 22], and for various healthcare professionals and students [21]. The scale showed positive attitude of healthcare professionals in various settings.

Previous studies with physiotherapy students investigated beliefs and attitudes towards elderly with other scales. In a convenience sample of 175 students in Scotland, Duthie and Donaghy [54] found that physiotherapy students’ attitudes towards older people were mainly neutral or positive using the Aging Semantic Differential questionnaire. They also observed some minor differences between first- and fourth-year students’ attitudes. Similarly, Bakırhan et al. [23] found that the attitudes and behaviour of 1270 physiotherapy students towards older people were positive. They identified stronger positive attitudes and behaviour in students who wanted to work in the field of geriatric rehabilitation after their graduation. Other studies published last decades also reported that physiotherapy students had neutral or positive attitude towards older people [55–57]. Generally, physiotherapy students showed rather positive attitude towards elderly people. As their scores varied between a neutral or positive attitude, it shows some room for intervention aiming at improving their attitudes. This should be a priority for educational institutions in order to improve the quality of care.

There may be differences across the health care disciplines in ageist attitudes [58]. Using the Kogan’s attitude towards older people (KAOP) scale, nursing students scored higher (139.12 ± 14.27) than students of other departments including physiotherapy (127.09 ± 9.87) [59] whereas in Turan et al. [56] with KAOP, the attitudes of physiotherapy students towards older people were better than students in other health disciplines (*p* < 0.05). Differences were also shown in Golden et al. [50] where nurse practitioner and social work students showed a stronger positive attitude towards elderly people than medical students did. Moreover, the nursing students had a higher perceived value of interprofessional healthcare of the elderly than medical students did. These studies showed that nurses and allied health students demonstrated more positive attitude towards elderly than medical students did. It may be related to differences among training and exposure with the patients.

In this work, study level and gender were assessed as potential factors that influence attitudes towards elderly people. Study level showed no significant difference between the three study years, even if there was a tendency to stronger positive attitude with increasing study level. In previous studies, significant differences between first-year and fourth-year medical students were reported [26], as well as differences between students and specialists [21] and between primary care residents and fellows [20, 22]. Regarding gender, conflicting results are reported in the literature. As in our work, three studies reported no effect of gender on attitudes towards elderly people among medical students [24], among medical resident and fellows [20] and among different health professionals and students [21]. However, women showed stronger positive attitudes than men did in a study with physiotherapy and rehabilitation students [23] and in a study with medical students [49]. Further factors were assessed in the literature. It was shown that an interest or intention to work in geriatrics was correlated with better attitude for physiotherapy and rehabilitation students [23], and for medical students [24, 26, 49]. Previous contact with elderly people also showed a positive correlation with the attitudes of physiotherapy and rehabilitation students [23, 54]. In addition, students who participated in relevant teaching also demonstrated a more positive attitude towards elderly people [23, 54]. Further factors, such as age and ethnicity, did not demonstrate any difference regarding the attitude of health care students or professionals [20, 49]. Finally, the anxiety about aging can also affect attitude towards older people. Indeed, students with high levels of anxiety about aging held more negative attitude towards older people than students with lower anxiety about aging [60]. Among all factors, it seems that personal experience and interest in a career in geriatrics, as well as training are correlated with a better attitude towards elderly people. Educational programme should provide adequate training to all healthcare professionals and support positive experience with elderly people.

This study reported positive attitudes of the physiotherapy students regarding exercise-related aging stereotypes with the ASES. Further, a significant change between physiotherapy students from the first and students from the second and third year was observed. This result suggests a change in the attitudes and stereotypes of students towards exercise and physical activity in older people with increasing study level. One hypothesis is that educational program influences the attitudes and behaviours of the students towards elderly people. The teaching program follows the recommendation from World Physiotherapy (WCPT) on physical activity and includes courses on physical activities for elderly people. In addition, physiotherapy students from the third year were encouraged to define physical activity program and they showed sensitivity to the different needs depending on the target population in their online physical activity program during the Covid-19 pandemic. However, we must be cautious, as this result does not originate from a longitudinal study. In the development and validation study of the ASES [28], the scale showed differences between younger and older adults, with older adults showing less stereotypes than younger ones. To the best of our knowledge, this study is the first one showing a change amongst different levels of physiotherapy students.

Several limitations of this study must be noted. First, the UCLA-GAS-F was tested only with undergraduate physiotherapy students on a single school in Switzerland. The participants represented a homogeneous group; they are young adults within the same educational programme. This homogeneous sample may affect the generalisability of the results. Indeed, the psychometric properties demonstrated in this study may change when assessed in other institutions or with other healthcare students and healthcare professionals. Second, the sample size (*n* < 200) was relatively small for a confirmatory factor analysis. It might have an effect on the fit indices as the small sample size might increase type I error (rejection of the model) [44]. Because CFA was not the primary objective of the study, this analysis might be underpowered. Thus, further studies are required to assess the structure of the UCLA-GAS. Third, the study design did not include longitudinal follow-up of the participants. Thus, it is not possible to assess attitude changes and to report responsiveness of the instrument. These aspects should be assessed in further study. Finally, to the best of our knowledge, no other tool in French is available to assess the same concept as the UCLA-GAS. Nor is there any gold standard available for the assessment of attitudes towards elderly people. Therefore, this study was limited for the evaluation of construct validity (discriminant and structural).

Although we cannot exclude a social desirability bias when undergraduate physiotherapy students completed questionnaires on attitudes towards older people and caring for older patients, their attitude was neutral to positive in this study. Several studies with students found that positive attitude towards the elderly was the main factor associated with a willingness to consider a career in geriatric medicine [24, 26, 61]. To provide quality care and treatment to the elderly, future health professionals must be prepared and willing to work with this population. However, few health students including in physiotherapy want to work in geriatrics [24, 61].

The availability of such a tool in French may be of interest and usefulness for people working with the elderly population in research and clinical contexts as well as for teaching institutions to assess the benefits of teaching materials on attitude.

## Conclusion

The UCLA-GAS was successfully translated and culturally adapted into French. The French version achieved good equivalence with the source version. The translated version was valid and reliable measure to assess general attitudes towards older people and caring for older patients. However, these issues need further empirical validation. The questionnaire is easily understandable and can be administered and completed by students and used in teaching institutions. Further studies are needed to evaluate responsiveness or sensitivity to change of the UCLA-GAS-F in a longitudinal cohort study of healthcare students.

## Supplementary Information

**Additional file 1.**

## Data Availability

The dataset during and/or analysed during the current study is available from the corresponding author on reasonable request.
