# Supplementary material for: Transcultural adaptation and validation of a French version of the University of California, Los Angeles geriatrics attitudes scale (UCLA-GAS-F)
Source: Arch Physiother. 2021 Sep 7;11:21. doi: 10.1186/s40945-021-00114-1 (PMC8423334; doi:10.1186/s40945-021-00114-1)
Supplement: Supplementary file 1 — Additional file 1. [file 40945_2021_114_MOESM1_ESM.docx]

French version of the University of California, Los Angeles Geriatrics Attitudes Scale (UCLA-GAS-F)

THE 14-ITEM GERIATRICS ATTITUDES SCALE – French version

INDICATIONS: Veuillez utiliser l’échelle pour indiquer dans quelle mesure vous êtes d’accord ou pas avec chaque énoncé. Il n'y a pas de bonnes ou de mauvaises réponses. La meilleure réponse est celle qui reflète vraiment votre opinion personnelle. Les résultats de cette étude seront rapportés uniquement par groupe, sans noms individuels identifiés. Les « personnes âgées » et les « patients âgés » mentionnés dans les questions se réfèrent aux personnes âgées de 65 ans et plus.

|  |  | Pas du tout d’accord | Plutôt pas d’accord | Incertain | Plutôt d’accord | Tout à fait d’accord |
| --- | --- | --- | --- | --- | --- | --- |
| 1. | La plupart des personnes âgées sont d’agréable compagnie. | 1 | 2 | 3 | 4 | 5 |
| 2. | Le gouvernement (fédéral) devrait réallouer des fonds de l’assurance-maladie des personnes âgées à la recherche sur le VIH ou les maladies pédiatriques. | 1 | 2 | 3 | 4 | 5 |
| 3. | Si j’ai le choix, je préfère voir des patients jeunes plutôt que des patients âgés. | 1 | 2 | 3 | 4 | 5 |
| 4. | Il est de la responsabilité de la société de fournir des soins aux personnes âgées. | 1 | 2 | 3 | 4 | 5 |
| 5. | Les soins médicaux aux personnes âgées mobilisent trop de ressources humaines et matérielles. | 1 | 2 | 3 | 4 | 5 |
| 6. | Lorsque les personnes vieillissent, elles deviennent moins organisées et plus confuses. | 1 | 2 | 3 | 4 | 5 |
| 7. | Les patients âgés ont tendance à être plus reconnaissants des soins médicaux que je leur prodigue que les jeunes patients. | 1 | 2 | 3 | 4 | 5 |
| 8. | Recueillir les antécédents médicaux auprès des personnes âgées est souvent pénible. | 1 | 2 | 3 | 4 | 5 |
| 9. | J’ai tendance à porter plus d’attention et éprouver plus de sympathie envers mes patients âgés qu’envers mes patients plus jeunes. | 1 | 2 | 3 | 4 | 5 |
| 10. | En général, les personnes âgées ne contribuent pas beaucoup à la société. | 1 | 2 | 3 | 4 | 5 |
| 11. | Le traitement des patients âgés atteints de maladies chroniques est vain. | 1 | 2 | 3 | 4 | 5 |
| 12. | Les personnes âgées ne contribuent pas de manière équitable aux coûts de leurs soins de santé. | 1 | 2 | 3 | 4 | 5 |
| 13. | En général, les personnes âgées agissent trop lentement pour la société actuelle. | 1 | 2 | 3 | 4 | 5 |
| 14. | Il est intéressant d’écouter le récit que les personnes âgées font de leurs expériences passées. | 1 | 2 | 3 | 4 | 5 |
